# Innovations in noninvasive sensory stimulation treatments to combat Alzheimer’s disease

**DOI:** 10.1371/journal.pbio.3003046

**Published:** 2025-02-28

**Authors:** Jung M. Park, Li-Huei Tsai

**Affiliations:** 1 Picower Institute for Learning and Memory, Massachusetts Institute of Technology, Cambridge, Massachusetts, United States of America; 2 Department of Brain and Cognitive Sciences, Massachusetts Institute of Technology, Cambridge, Massachusetts, United States of America; 3 Broad Institute of MIT and Harvard, Cambridge, Massachusetts, United States of America

## Abstract

Alzheimer’s disease (AD) is a progressive neurodegenerative disorder affecting millions worldwide. There is no known cure for AD, highlighting an urgent need for new, innovative treatments. Recent studies have shed light on a promising, noninvasive approach using sensory stimulation as a potential therapy for AD. Exposing patients to light and sound pulses at a frequency of 40 hertz induces brain rhythms in the gamma frequency range that are important for healthy brain activity. Using this treatment in animal models, we are now beginning to understand the molecular, cellular, and circuit-level changes that underlie improvements in disease pathology, cognition, and behavior. A mechanistic understanding of the basic biology that underlies the 40-hertz treatment will inform ongoing clinical trials that offer a promising avenue of treatment without the side effects and high costs typically associated with pharmacological interventions. Concurrent advancements in neurotechnology that can also noninvasively stimulate healthy brain rhythms are illuminating new possibilities for alternative therapies. Altogether, these noninvasive approaches could herald a new era in treating AD, making them a beacon of hope for patients, families, and caregivers facing the challenges of this debilitating condition.

## Abstract 2

Current treatments for Alzheimer disease are hampered by limited efficacy, side effects and high costs. This Essay highlights non-invasive sensory stimulation as a promising emerging technology for the treatment of Alzheimer disease.

## Introduction

Historically, treatment of Alzheimer’s disease (AD) has focused on managing symptoms rather than addressing the disease’s underlying causes. Dr. Alois Alzheimer first reported on the disease in the early 1900s, and we have since discovered two distinct neurological hallmarks of AD: amyloid beta plaques and tau neurofibrillary tangles [1]. However traditional therapies that target these hallmarks have yielded modest results. Meanwhile, our limited biological understanding of neurodegeneration has shaped our healthcare system to primarily address the visible cognitive and behavioral manifestations of the disease. By the time patients are properly diagnosed, they usually exhibit clear signs of dementia, including loss of memory, executive function, and motor control. Retroactive treatments like surgeries and implants are prone to complications and seldom lead to long-term health improvements [2]. Noninvasive interventions, including cognitive therapy [3], physical exercise [4], and dietary modifications [5], have had limited impact on slowing disease progression or are more effective as preventive measures [6]. This symptomatic approach underscores the urgent need for therapies that can target the fundamental biological processes underlying AD pathogenesis.

Recent AD drugs, despite representing significant scientific progress, have faced various issues and limitations. Treatments recently approved by the US Food and Drug Administration, such as aducanumab [7,8] and lecanemab [9], have sparked debate due to their high costs, modest efficacy, and potential side effects. These drugs have been shown to markedly clear amyloid beta plaques in the brain and slow cognitive decline by 27%–35% following 18 months of treatment. However, the drugs are also associated with undesirable side effects such as brain swelling and bleeding in 40% of patients treated with aducanumab [10,11]. Overall, the complexity of AD, in which multiple brain regions, circuits, cell types, and molecular pathways are compromised, means that targeting a single mechanism often falls short of delivering comprehensive benefits. These limitations underscore the urgent need for a more robust, multifaceted, and comprehensive treatment that can effectively combat this devastating condition.

**Fig 1:**
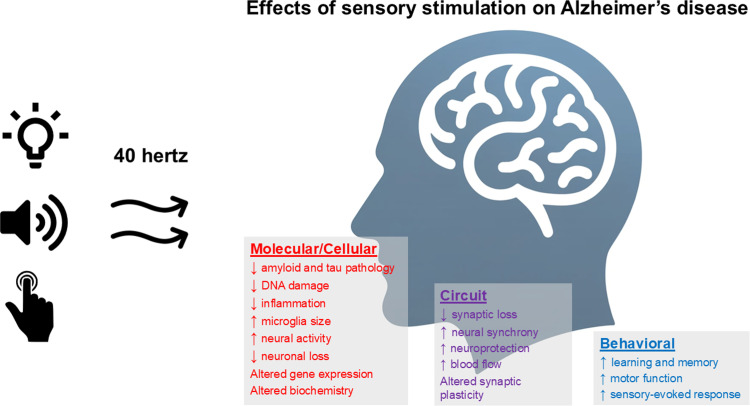
Audio, visual, and tactile 40-hertz sensory stimulations induce changes at molecular, cellular, and circuit levels. Using sensory stimuli, we can noninvasively promote regions of the brain to oscillate at a 40-hertz frequency in both mouse models [12,14,15] and human patients with AD [13,16,17]. This in turn produces multifaceted changes that promote improvements in cognitive and motor capabilities.

In an effort to overcome these limitations, our research group developed a noninvasive treatment for AD that stimulates affected brain regions at 40 hertz (40 cycles per second) [12]. These stimulations can promote the brain to oscillate at this same frequency and drive holistic functional changes at molecular, cellular, and circuit levels to improve disease pathology, cognition, and behavior in animal models of AD. Early clinical evidence shows therapeutic potential in human patients (Fig 1) [13]. In this Essay, we discuss evidence showing that promoting 40-hertz gamma band activity in the brain can provide an effective, accessible treatment option that targets neuronal, glial, and vascular networks that are compromised in AD. We delve into the known and unknown underlying mechanisms by which 40-hertz stimulation elicits these effects. We examine the benefits and limitations of these treatments, concluding that 40-hertz stimulation offers an innovative and promising avenue for the future of AD treatment.

### Why brain rhythms matter

The human brain is an incredibly complex organ composed of ~86 billion neurons and trillions of synapses [18]. Each neuron can form thousands of synapses, creating vast and intricate circuits that enable transmission of information across various brain regions. Research has shown that, in turn, the brain communicates through rhythmic patterns of neural activity that synchronize to form coherent networks. These oscillations, particularly in the gamma frequency range (30–100 hertz), are crucial for higher-order functions such as integrating sensory information, attention, perception, and memory [19]. As such, disruptions in these rhythmic patterns are implicated in many neurological and psychiatric disorders such as schizophrenia [20], epilepsy [21], autism [22], and AD [19].

In AD patients, the accumulation of toxic amyloid proteins and other disease pathologies disrupts brain signaling and impairs neuronal synchronization across brain regions [23]. This includes altered slow gamma rhythms (30–50 hertz), which are documented in human patients and animal models [24–26]. Promoting healthy oscillations in the gamma range is therefore a promising target for therapeutic interventions [27].

### Optogenetics to induce gamma oscillations in mice

Optogenetics is a groundbreaking technique that utilizes light to control the electrical activity of neurons genetically modified to express light-sensitive ion channels [28]. This allows precise manipulation of neuronal activity, enabling researchers to study the functions of specific neural circuits and their roles in behavior and disease [29]. In 2009, we demonstrated that optogenetics can be used to drive gamma oscillations in the somatosensory cortex of mice [30]. More recently, by optogenetically activating hippocampal neurons at 40-hertz, we successfully induced hippocampal gamma oscillations in an amyloid beta mouse model of AD. This treatment altered morphology of brain immune cells called microglia and markedly reduced amyloid beta levels [30].

Notably, another group independently demonstrated an alternative approach to restore gamma waves in a different amyloid-based mouse model of AD. Here, overexpressing the sodium channel subunit Nav1.1 (which is reduced in AD) increased gamma oscillations in the cortex and improved cognitive function in the animals [31]. These early findings provided compelling evidence that gamma oscillations can fundamentally alter molecular and cellular biology, thereby influencing disease pathology and behavior.

### Noninvasive sensory stimulations to induce gamma oscillations in mice

Optogenetics, while promising and powerful, are invasive and more difficult to translate to human patients [32,33]. Fortunately, it was demonstrated that brain waves can be manipulated in cats using only visual stimulations to the retina [34]. Inspired by this, our team pioneered the use of noninvasive technique called Gamma ENtrainment Using Sensory stimuli technique (GENUS), which uses sensory stimulations to deliver gamma oscillations to the brains of AD model mice.

Indeed, exposing mice to 40-hertz flickering lights, referred to as visual GENUS, induced gamma oscillations in the visual cortex – a brain region that processes visual information. Implementing a more extensive one-hour daily protocol over the course of a week activated microglia and reduced amyloid levels in the visual cortex. This procedure also reduced levels of hyper-phosphorylated tau protein in neurons [12], another hallmark of AD [35].

In a follow-up study, we discovered that visual GENUS drives gamma oscillations far beyond the visual cortex. Gamma activity propagated to the hippocampus and prefrontal cortex, regions essential for learning, memory, and higher cognitive functions such as decision-making and working memory. Chronic GENUS treatment (one hour per day for several weeks) before the onset of neurodegeneration preserved neuronal and synaptic density in all three brain regions from the adverse effects of tau protein and neurotoxic protein overexpression. This neuroprotection translated to improved memory performances across multiple mouse models of neurodegeneration [36].

The GENUS stimulation protocol has since been further refined to enhance the benefits of sensory-evoked gamma oscillations in the brain. Using 40-hertz tones in mouse models that are predisposed to developing AD symptoms, we successfully induced gamma oscillations in the hippocampus and auditory cortex, a brain region that processes sound. A one-hour daily exposure to auditory GENUS over the course of a week significantly reduced amyloid beta and tau pathology in both brain regions and improved memory performances. Crucially, auditory GENUS not only activated microglia but also increased vascular dilation in the brain, potentially facilitating the clearance of amyloid plaques by increasing brain blood flow [14]. Promisingly, combining 40-hertz visual and auditory stimulations in the same AD mouse models proved more effective than either modality alone. Microglia clustered in greater numbers around amyloid plaques, and we observed reduced plaque levels in a larger portion of the brain including the prefrontal cortex [14].

More recently, inspired by the effects of audiovisual GENUS, we developed a tactile GENUS system to deliver 40-hertz vibrations to mouse models of neurodegeneration. The somatosensory and motor cortices, which process our sense of touch and produce movement commands for the body, respectively, are highly interconnected. These regions are affected early in individuals with AD, with motor decline often preceding cognitive symptoms of the disease [37]. When we delivered tactile GENUS to a tauopathy mouse model that recapitulates frontal temporal dementia and progressive supranuclear palsy pathology, we observed lower levels of phosphorylated tau, neuronal and synaptic loss, and DNA damage in the somatosensory and motor cortices. Such decreases were accompanied by increased neural activity in those brain regions as well as improved motor abilities [15].

These studies provide encouraging evidence that promoting gamma oscillations produces multifaceted effects at molecular, cellular, and circuit levels to drive improvements in cognition and behavior (Fig 1). Such alterations, especially to the molecular and cellular pathways, suggest that the improvements observed with GENUS are more than just an epiphenomenon. Indeed, GENUS treatment shows great promise in treating neurological and psychiatric disorders beyond AD (see Box 1). However, the basic biological mechanisms by which the beneficial effects arise have remained largely unknown. What is driving microglia to change their gene expression profile? What is preventing neuronal, synaptic, and DNA damage? And what is driving amyloid clearance throughout the brain?

**Box 1. 40-hertz sensory stimulation to treat neurological and psychiatric disorders.**

The multifaceted neuroprotective effects of GENUS, from reducing DNA damage and inflammation to increasing brain activity, show promise in treating a variety of conditions other than AD. The treatment’s far-reaching consequences are likely driven in part by the fact that gamma entrainment propagates to brain regions far beyond areas impacted by AD (i.e., hippocampus). In our lab, audiovisual GENUS protected mice from chemotherapy-induced impairments such as decreased brain volume, DNA damage, inflammation, and cognitive deficits [38]. In a mouse model of multiple sclerosis – as characterized by the loss of myelin that forms around neurons to facilitate effective communication between cells – audiovisual GENUS mitigated against demyelination and preserved the functional integrity and plasticity of long-range connections that link the two brain hemispheres [39]. Ongoing work is looking at GENUS effects in mouse models of Parkinson’s disease, Down syndrome, and gut microbiome with age. Other groups have also shown promising efficacy of 40-hertz sensory stimulation in conferring neuroprotective effects against a host of disease states. For example, visual GENUS conferred neuroprotection after ischemic injury [40] and reduced anxiety susceptibility in post-stroke mice [41]. Audiovisual GENUS improved cognitive and motor symptoms, as well as reversing depressive-like behavior in a Parkinson’s mouse model [42]. The multimodal treatment reduced interictal epileptiform discharges in human focal epilepsy patients [43]. Others have even proposed using GENUS as a nonpharmacological therapeutic intervention for schizophrenia [44]. The versatility of GENUS reflects the multifaceted neuroprotective effects of the treatment.

### Uncovering the biological mechanism of gamma treatment

**Fig 2:**
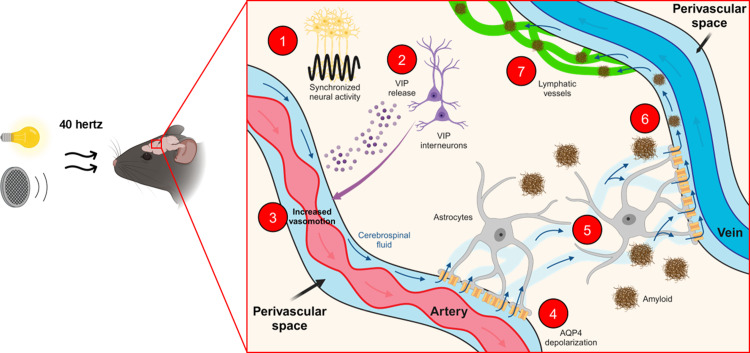
40-hertz sensory stimulation promotes clearance of amyloid plaques in the brain through the glymphatic system. We are now starting to understand the basic biological mechanism(s) by which the improvements in AD pathology arise following GENUS treatment. (1) Audiovisual GENUS synchronizes neural activity to oscillate at 40-hertz. (2) This promotes vasoactive intestinal polypeptide-expressing (VIP) interneurons to release VIP neurotransmitters. (3) These proteins in turn cause rhythmic contraction and relaxation of vascular smooth muscles of arterioles and (4) the increased depolarization of aquaporin-4 proteins (AQP4) at astrocytic endfeet where the astrocytes form contacts with blood vessels. AQP4 depolarization enables the (5) influx of cerebrospinal fluid from the perivascular space that surrounds blood vessels to the brain, allowing (6) amyloid to be washed away and (7) drained to meningeal lymphatic vessels. The activation of the waste-clearing glymphatic system is likely one of many mechanisms by which GENUS confers neuroprotection against Alzheimer’s disease pathology [46]. The figure was partially created with BioRender.com.

One mechanism by which 40-hertz oscillations are thought to aid amyloid clearance is through stimulation of the glymphatic system, which controls the flow of cerebrospinal fluid to clear waste from the brain [45]. Synchronized gamma rhythms in the brain prompt inhibitory neurons called vasoactive intestinal polypeptide-expressing (VIP) interneurons to release VIP neuropeptides [46]. This aligns with previous studies demonstrating that neuropeptide release is facilitated by high-frequency neural activity [47]. These proteins in turn increase arterial vasomotion and the polarization of aquaporin 4 water channels in astrocytes (neuron-supporting glial cells). Such changes promote amyloid clearance via the brain’s glymphatic system [45]. Treating AD mice with audiovisual GENUS increased both cerebrospinal fluid and blood flow in the brain to clear amyloid plaques [46] (Fig 2). Crucially, another group independently demonstrated that visual GENUS alone can activate adenosine signaling to promote glymphatic clearance in wild-type mice [48]. These results provide a mechanistic framework by which 40-hertz brain rhythms promote neuropeptide release by interneurons to facilitate the glymphatic clearance of amyloid proteins [46].

Due to the multifaceted effects on molecular and cellular pathways induced by GENUS, glymphatic clearance of amyloid is likely not the only underlying mechanism at play. For example, we discovered that visual GENUS boosts expression of genes associated with DNA repair, synaptic function, and biochemical processes critical for synaptic communication in neurons; microglia also alter their gene expression profiles by decreasing expression of inflammation-promoting genes [36]. In parallel, visual GENUS is also being used to investigate how gamma oscillations increase levels of immune signal proteins called cytokines to rapidly recruit microglia [49,50]. While much progress has recently been made, it is important to recognize that the precise mechanisms underpinning the beneficial effects of gamma sensory stimulation in ameliorating AD pathology are not yet fully understood. For example, the glymphatic system also clears tau via the aquaporin 4 channels [51,52], but does GENUS similarly mediate tau clearance through VIP release? We also do not yet know how GENUS improves cognitive function. Recent papers suggest multiple contributing factors such as enhanced neural synchronization to low gamma frequencies in the hippocampus, increased synaptic density in the hippocampus and prefrontal cortex, increased white matter volume, and improved oligodendrocyte survival following GENUS treatment [36,38,39]. Further research is required to establish a mechanistic understanding of GENUS so that we can ultimately guide the development of therapeutic treatments for human patients.

### Benefits of gamma treatment in Alzheimer’s disease patients

Neuroscientists often lament that it is a great time to have AD if you are a mouse. Pharmaceutical drugs that showed great promise in animal models have often failed to deliver results in human clinical trials [53,54]. The few that recently passed Food and Drug Administration approval are promising, but there is a general consensus that more effective and safer treatment options are needed. Our ultimate goal, therefore, is to translate GENUS discoveries into a safe, accessible, and noninvasive therapy for AD patients.

With this in mind, we created an audiovisual GENUS device for humans that is safe and can successfully induce gamma oscillations in cortical regions and deeper brain structures such as the hippocampus and amygdala [16]. In an early clinical trial, three months of daily treatment reduced brain atrophy – including in the hippocampus – and strengthened functional connectivity as measured by synchronization across brain regions. Patients also showed promising cognitive improvements. These findings demonstrate that 40-hertz sensory stimulations can produce clinical benefits in AD patients at least in the short term [16]. Ongoing research is being conducted to study the long-term effects of audiovisual GENUS, as well as exploring therapeutic potential of tactile GENUS to improve motor function as observed in animal experiments [15].

Inspired by early patient results, large-scale clinical trials are underway to optimize the therapeutic applications of GENUS. One company, Cognito Therapeutics, developed a medical audiovisual stimulation device deemed safe and well-tolerated by AD patients. Results from daily 1-hour treatments for 6 months – such as a 69% reduction in brain volume loss – are promising [17]. This includes reduced atrophy in the corpus callosum, a brain region critical for interhemispheric communication and cognitive function [55]. Cognito Therapeutics has received Food and Drug Administration approval to conduct stage III clinical trials using their medical device. Critically, other research groups have replicated [56] and corroborated the health benefits of 40-hertz sensory stimulations [55,57–59]. For example, 40-hertz stimulation improved sleep in both AD mouse model and human patients [57,60].

In addition to sensory methods, electrical and magnetic stimulations have recently demonstrated efficacy in noninvasively inducing 40-hertz brain activity in mice and human patients (Box 2) [61,62]. Both methods drove a host of improvements in AD patients, such as decreases in brain volume loss, amyloid and tau pathology, and functional connectivity. While the use of electromagnetic brain stimulations as a potential treatment for AD is in its early stages, the techniques provide a promising noninvasive alternative to sensory stimulation methods.

**Box 2. Noninvasive electromagnetic approaches to treat Alzheimer’s disease.**

Transcranial alternating current stimulation delivers electric currents from electrodes that are placed on a patient’s scalp. Similarly, transcranial magnetic stimulation delivers magnetic pulses to induce electrical currents in specific regions of the brain. Both techniques can promote gamma oscillations in the brains of both animal models and AD patients. 40-hertz electromagnetic treatments increase memory, executive functions, global cognition, functional connectivity, and cerebral blood flow in AD patients. These changes are accompanied by decreases in grey matter loss, tau, and amyloid pathology in the brain [13,63]. While the implementation of these technologies in human patients is in the early stages, electromagnetic treatments provide alternative intervention options that hold great promise in conferring neuroprotection against AD pathology. Unlike GENUS, electromagnetic interventions enable targeted treatments of specific brain regions. But as with GENUS, further work is required to determine the basic biological mechanisms by which gamma stimulation translates to beneficial effects.

While encouraging, it is essential to note that no clinical trials have demonstrated that GENUS or any other treatment can cure or reverse the progression of AD. One likely explanation is that most clinical studies investigated the effects of GENUS in patients already exhibiting disease neuropathology and cognitive impairments [13,16,58,64]. As with federally approved AD drugs, GENUS may be most effective when delivered during earlier stages of the disease, or even as a preventive therapy for some people with a genetic predisposition to the disease. It will be worthwhile to further access the treatment in patients with mild cognitive impairments or even in patients at risk who do not yet show AD pathology.

### Potential limitations of gamma stimulation therapy

A potential limitation of gamma stimulation treatments is the methodological variability across various clinical studies. Although sensory stimulation devices are cost-effective and can easily be assembled by individual labs across the world, this also means that research groups differ in their delivery of the treatment. For example, some labs use portable monitors with speakers [16] while many larger clinical trials use wearable headsets to deliver audiovisual stimuli [13,17,55]. There is also variability in the intensity of the stimuli, as measured by the lumens and decibels of light and sound, respectively. Furthermore, groups have independently reported that different light wavelengths differentially enhance gamma brain activity [65–67]. It may benefit the field to establish standardized guidelines while still optimizing the parameters so that we can both ensure better comparisons across the various studies and continued improvements to the system.

Another possible drawback of an at-home GENUS device is the difficulty in maintaining patient compliance. Pharmaceutical drugs like lecanemab and donanemab are intravenously administered by trained health professionals. Oral medications like donepezil are easily consumable and have high adherence rates among patients. However, GENUS requires patients to self-administer the 1-hour daily stimulation protocol, making it difficult to ensure consistent and correct use. Ultimately, however, we believe that the affordability and flexibility of the at-home portable GENUS devices are major attributes that outweigh any potential drawbacks.

Finally, many of the early clinical studies are underpowered and can benefit from larger trials with longer treatment durations. For example, one electrical stimulation study demonstrated significant decreases in tau pathology in three out of five patients, but no changes in amyloid beta levels in any of the patients [68]. Many of the early electromagnetic stimulation trials also lacked placebo conditions. This makes it difficult to draw definitive conclusions about their 40-hertz treatment [69,70]. Meanwhile, stage III clinical trials with large patient enrollment and proper control groups are underway to better illuminate the beneficial effects of GENUS in treating AD.

A comprehensive summary of the clinical findings using noninvasive electromagnetic or sensory stimulations to treat Alzheimer’s disease can be found in a recent review article [13]. The field of noninvasive treatments for AD is still in its early stages, and further research is required to translate laboratory findings to clinical populations. But it is already abundantly clear that despite the present constraints, noninvasive 40-hertz gamma stimulation techniques demonstrate the therapeutic potential of engaging our own brain rhythms to fight against AD.

## Conclusions and future directions

As lifespans increase and our population ages, AD poses a significant public health challenge with substantial societal costs [6]. While scientists have made tremendous progress in our fundamental understanding of AD pathology, we have had limited success in translating our laboratory findings into clinical results [53,54]. However, recent advances in neurotechnology have heralded a new age in combating this neurodegenerative disease.

Breakthroughs in diagnostic tools, including advanced imaging techniques and disease biomarkers [6,71–73], enable earlier and more accurate detection of AD long before the appearance of cognitive symptoms. Next-generation sequencing and genomic techniques have identified novel therapeutic targets that have launched new avenues of research [74–76]. Finally, open-source tools [77–79] have fostered the development of affordable, customizable hardware and software, making cutting-edge neurological research and applications more accessible to a wider audience. This has enabled the development and widespread use of GENUS to be studied by groups all over the world.

Since we first pioneered the use of GENUS to induce gamma rhythms in the brain, multiple research groups have replicated the findings and contributed to our mechanistic understanding of the treatment’s biological mechanisms [56,60,80]. At the molecular and cellular level, we are beginning to understand how gamma oscillations can promote amyloid clearance via the glymphatic system (Fig 2). At the circuit level, GENUS promotes robust rhythmic firing in the corresponding sensory cortices. Critically, gamma oscillations synchronize to other regions important for learning, memory, and high-order functions such as the hippocampus and prefrontal cortex. We also find decreased synaptic loss coupled with increased neuroprotection and blood flow [12,14,15,36].

It is important to note that one group found limited effects of GENUS in inducing native gamma rhythms in the hippocampus and amyloid plaque levels in mouse models of AD [81]. However, methodological differences may have contributed to the contrasting conclusions [82]. For example, this study did not observe amyloid reduction following 40-hertz sensory stimulation, possibly due to pooling of animals from multiple AD mouse models, sexes, and ages. The experiments also lacked a positive control, and the authors processed paraformaldehyde fixed tissue for a sensitive enzyme-linked immunosorbent assay [82,83]; fixed tissues for such assays are not recommended for multitude of reasons such as loss of enzyme activity, limited antigen extraction, increased background noise, and reduced sensitivity.

Overall, the comprehensive benefits of the noninvasive 40-hertz sensory and even electromagnetic treatments (see Box 2) – from altering immune cell responses to promoting glymphatic clearance – may underlie the interventions’ wide-ranging effectiveness in ameliorating AD pathology. However, further work is required to better understand how neuronal activity produced by gamma oscillations translates to beneficial effects across the brain. For example, we still do not understand how gamma synchronization propagates beyond the sensory cortices. A mechanistic understanding of gamma propagation will allow clinicians to provide personalized care by enhancing the spread of gamma to targeted brain regions that are selectively impaired in individuals.

After decades of limited progress, advances in neurotechnology have ushered in a new era in the treatment of AD. The advent of noninvasive 40-hertz sensory stimulation treatment has brought renewed optimism to patients, caregivers, and researchers alike. As these innovative therapies continue to develop, they hold the promise of significantly improving outcomes and quality of life for those affected by this devastating condition.
